# Anomaly Detection Module for Network Traffic Monitoring in Public Institutions

**DOI:** 10.3390/s23062974

**Published:** 2023-03-09

**Authors:** Łukasz Wawrowski, Andrzej Białas, Adrian Kajzer, Artur Kozłowski, Rafał Kurianowicz, Marek Sikora, Agnieszka Szymańska-Kwiecień, Mariusz Uchroński, Miłosz Białczak, Maciej Olejnik, Marcin Michalak

**Affiliations:** 1 Łukasiewicz Research Network—Institute of Innovative Technologies EMAG, ul. Leopolda 31, 40-189 Katowice, Poland; lukasz.wawrowski@emag.lukasiewicz.gov.pl (Ł.W.); andrzej.bialas@emag.lukasiewicz.gov.pl (A.B.); artur.kozlowski@emag.lukasiewicz.gov.pl (A.K.); rafal.kurianowicz@emag.lukasiewicz.gov.pl (R.K.); marek.sikora@polsl.pl (M.S.); 2 Wroclaw Centre for Networking and Supercomputing, Wroclaw University of Science and Technology, Wybrzeże Wyspiańskiego 27, 50-370 Wrocław, Poland; adrian.kajzer@pwr.wroc.pl (A.K.); agnieszka.kwiecien@pwr.edu.pl (A.S.-K.); mariusz.uchronski@pwr.edu.pl (M.U.); milosz.bialczak@pwr.wroc.pl (M.B.); m.olejnik@pwr.edu.pl (M.O.); 3 Department of Computer Networks and Systems, Silesian University of Technology, ul. Akademicka 16, 44-100 Gliwice, Poland

**Keywords:** anomaly detection, cybersecurity, network traffic monitoring

## Abstract

It seems to be a truism to say that we should pay more and more attention to network traffic safety. Such a goal may be achieved with many different approaches. In this paper, we put our attention on the increase in network traffic safety based on the continuous monitoring of network traffic statistics and detecting possible anomalies in the network traffic description. The developed solution, called the anomaly detection module, is mostly dedicated to public institutions as the additional component of the network security services. Despite the use of well-known anomaly detection methods, the novelty of the module is based on providing an exhaustive strategy of selecting the best combination of models as well as tuning the models in a much faster offline mode. It is worth emphasizing that combined models were able to achieve 100% balanced accuracy level of specific attack detection.

## 1. Introduction

The paper presents the results of the RegSOC project in the range of anomaly detection. The aim of the project was to establish methods and tools dedicated to small and medium-sized enterprises and public institutions to detect anomalies in their computer networks. The paper presents a proof-of-concept for a created solution.

RegSOC is a specialized security operations centre (SOC), dedicated mainly to public institutions, developed according to the Polish cybersecurity strategy. RegSOC, similarly to other SOC systems, is a centralized organization unit embracing three pillars: people, technology, and processes.

The first pillar, people, includes highly qualified cybersecurity personnel of different competencies working inside the proper organizational structure and are able to permanently improve their skills and knowledge in the realm of technological progress, emerging attack methods, and IT users’ behaviours.

The technological pillar embraces advanced software and hardware solutions for security monitoring, network infrastructure readiness, event collections, correlation and analysis, security control, log management, vulnerability tracking and assessment, communication, threat intelligence, and many others. The key element is the SIEM (security information and event management) system.

The third pillar consists of SOC processes, run by SOC personnel with the use of technological equipment. Examples of SOC processes are security monitoring, security incident management, threat identification, digital forensics and risk management, vulnerability management, security analysis, etc. Some of them are internal, some are engaged directly in security services provided for SOC customers.

The SOC organization, processes, and technology are discussed in many publications, including the [1,2] books.

The RegSOC project is aimed at the development of the SOC prototype, which can be implemented several times in a cybersecurity infrastructure. The project research is related to:

- the procedural and organizational model of operation of the regional centres in cooperation with the national cybersecurity structure (people, processes);
- the cybersecurity monitoring platform (technology).

The paper is focused on the hardware and software equipment working as network-based intrusion detection systems (NIDS), able to operate as stand-alone autonomous devices within a local administration domain, as well as a device integrated with RegSOC. This NIDS is designed to monitor network traffic between internet service providers and the network infrastructures of customers (public, commercial) to detect different kinds of cyber attacks, i.e., signature-based and non-signature based.

Signature-based detection is a process where a unique identifier is created for a given known threat. This way the threat can be identified in the future by a virus scanner or IDS. Signatures are defined to look for characteristics within network traffic, e.g., Snort rules [3]. Generally, the attack is known and the protection method, which should be applied, is known. Most cyber-attacks are detected by this kind of method, but currently, they are not sufficient for security.

The security problem increases when a vulnerability is explored by an attacker who immediately applies a new attack method based on this vulnerability. It is identified as a zero-day attack. There are no signatures for such a threat and in this case, methods based on behavioural analysis should be applied. The network behaviour may indicate the attack symptoms. The network behaviour (traffic) can be “typical” or “atypical” with anomalies. This paper concerns the network traffic investigation to detect anomalies, which may indicate the symptoms of an attack. To detect anomalies and properly interpret their meaning, the machine learning approach is used.

Some traffic anomalies may be caused by atypical network users’ operations (software updates, transferring big files), and some may be symptoms of a known or previously unknown attack. This paper concerns these methods and is closely related to the anomaly detection module developed in the RegSOC project.

This paper is organized as follows: it starts with a short description of the whole consortium motivation to develop a module dedicated to outlier detection; later a more formal trial of anomaly definition approaches is presented, together with a brief review of existing anomaly detection approaches that finally led us to selecting two models of anomaly detection to be used in the module; afterwards, the anomaly detection module (ADM) with its software and hardware aspects is presented; the next part of this paper focuses on the application of ADM in a real network traffic analysis, i.e., which the traffic contained well-known (in time) attacks in the network, and the results of this application are extensively analysed, explained and discussed. The discussion focuses on a balanced accuracy-based comparison of the single models as well as on an exhaustive approach built from more complex meta-models; the paper ends with some conclusions and drafts of further work related to future ADM development.

## 2. Motivation

The evolution of cyber-attacks means that the problems a cybersecurity centre must solve are constantly new and frequently changing [4]. Therefore, we sought an approach to support the discovery of new phenomena in the IT environment as an essential component of the designed comprehensive protection system [5]. Threat detection methods based on anomaly detection seemed appropriate for this purpose, as they focus on identifying and assessing abnormal events and states [6,7,8]. At that time, research papers described anomaly detection techniques used, for example, to identify fraud [9,10], detect threats from internal users [11], or detect advanced DDoS attacks [12,13]. In the course of these studies, researchers adopted various approaches, such as statistical analysis, methods of classification and clustering, fuzzy sets, approximate inference, neural networks, and other hybrid techniques. The above solutions achieved satisfactory theoretical results, but when we started the project, the existing security systems did not use most of them. One of the reasons for this is that the use of historical datasets during research do not contain data relevant to current cybersecurity issues. The second is due to the problem of IT environment variability and its heterogeneity. These are natural features of modern IT systems, but their high dynamics make it challenging to diagnose atypical events, in particular in the context of security.

Therefore, during the project, we wanted to develop or adapt anomaly detection methods [14,15,16] that would consider the variability of the monitored network, recent data on threats and security incidents, and dependencies between the objects registered in the data. By assumption, these were to be interdisciplinary works, using knowledge in information processing (from statistics and data analyses to artificial intelligence), user behaviour in the IT system (social engineering, computer forensics), and the technical aspects of information systems and networks. We aimed to build an adequately tailored profile of the protected IT environment, using large datasets from simulated and real networks containing actual observations. An essential aspect of our work was developing a prototype one could use in a real security system. We decided to implement an anomaly detection module that could operate autonomously and integrate with the SIEM class system. The latter made it possible to correlate the results of the anomaly detection module with events of a different nature originating from other data sources.

## 3. Related Works

### 3.1. The Definition of an Anomaly

It is not possible to provide one strict definition of an anomaly. We rather “feel” what we want to express with such a word; however, a formal definition of an outlier changes as different approaches try to find them in the data.

During the last decade several working definitions of anomaly/outlier have been proposed by different scientists. Since the 1950s, the following attempts at outlier definition have been proposed in [17]: “an outlying observation is one that appears to deviate markedly from other members of the sample in which it occurs”. The author also tried to provide two possible causes of the outlier appearance: an extreme manifestation of the random variability inherent in the data or the result of the gross deviation from the prescribed experimental procedure (or an error in data acquisition).

Later, in [18] another descriptive definition of an outlier was provided: “An outlier is an observation that deviates so much from the other observations as to arouse suspicions that it was generated by a different mechanism”. Such an approach is quite close to one of the above-mentioned causes of outlier appearance. The Barnett and Lewis definition [19] is also quite comparable: that an outlier is an observation (or subset of observations) which appears to be inconsistent with the remainder of that set of data.

The key word “inconsistency” seems to be significant for Weisberg [20], who claimed that an outlier is a case that does not follow the same model as the rest of the data. This means that the outlier presence may come from the fact that there are observations in the data that come from two different well-defined models and the number of each model representative is not comparable (one model samples surpass the other one). That leads to the common issue of imbalanced data analysis in the classification tasks. In the paper [21] we may find the incorrect class labelling-based definition of an outlier: an object from class A is incorrectly assigned to class B, so from the B class point of view it can be treated as an outlying observation.

It is also worth mentioning the interpretation-based definition of an outlier’s nature [22]: one group as observations lying outside the data but interpretable as noise and a second as observations lying outside the data and noise. More comparable proposals of the outlier definition may be found in [23].

### 3.2. Anomaly Detection Approaches

In the paper ref. [24] outlier detection methods were divided into six categories:

- statistical-based methods (e.g., [25,26,27,28]);
- distance-based methods (e.g., [16,29,30]);
- density-based methods (e.g., [14,15,31]);
- clustering-based methods (e.g., [32,33];
- graph-based methods (e.g., [34]);
- ensemble-based methods (e, g. [35,36]);
- and learning-based methods (e.g., [37,38,39]).

Below, a short description of each mentioned group will be provided.

#### 3.2.1. Statistical-Based Methods

As a naive statistically based method of outlier detection in one-dimensional data with a normal distribution, a simple 3σ criterion can be mentioned: a value that differs from the mean value by more than three standard deviations is considered an outlying one. A more sophisticated approach is Grubb’s test [40] that may be one- or two-sided.

Another example of an statistical approach is the Gaussian mixture model [41]. In the paper [42] such an approach was used to detect anomalies in the phasor measurement units (PMU) of multivariate streaming. On the other hand, in the paper [43] a linear regression was used to detect anomalies. Furthermore, kernel density estimation-based methods were used as outlier detection approaches [44]. In the paper [45] adaptive Gaussian kernel widths were used in two aspects: a larger one to smooth the input data in high-density regions and a smaller one to detect anomalies in low-density regions.

#### 3.2.2. Distance-Based Methods

In such an approach, distances between all objects become the criterion for detecting anomalies. Most applications are based on the k-nearest neighbour and the distance threshold idea [46]. In the paper [47] a modification of such an approach was presented, based on dynamic programming. The further extension of such an approach may be also found in [48]. Apart from the above-mentioned applications, a kNN-based approach was successfully applied in [49,50].

#### 3.2.3. Density-Based Methods

Density-based methods make use of the following observation: an object should be interpreted as an anomaly when its local density (of other objects) is different from the local density of its neighbours. One of the most popular methods that is based on such an approach is the local outlier factor [14]. However, based on the above-mentioned algorithm a variety of modifications have also been developed [51,52] or [53].

#### 3.2.4. Clustering-Based Methods

Clustering means the search for groups of objects closer to each other than to objects from other groups and the found partition may become the input for another analytical task, just to mention classification. However, it is easy to change the interpretation of the clustering results in such a way that very small groups (or objects not assigned to any group) can be interpreted as outliers.

A density-based clustering algorithm—called DBSCAN [32]—allows to assign some objects to any group. This means that such an object is so far from others that it is impossible to interpret it as a member of any of the found groups. This means that it should be interpreted as a kind of outlying observation. Such an interpretation was extended also in other works, [54] or [55].

#### 3.2.5. Graph-Based Method

Sometimes it is difficult to provide a multidimensional description of objects while it becomes easier to map the dependencies between them with a graph. In [56], the following example is provided: when analysing reviewer-reviewed product data it may become much more important to represent the connections between the reviewer and the product in a graphical way and later to analyse the connections. Based on such an approach, a variety of methods have been developed [57,58,59].

#### 3.2.6. Ensemble-Based Methods

The ensemble approach is very popular in many applications, especially in classification [60], in particular AdaBoost [61] or random forests [62]. With such an idea not one but multiple models are built on training samples and the answer of any of them is taken into consideration to make the final decision. Accordingly, a set of separated anomaly detectors may be used for better results of outlier analysis. Such an approach was used in [63] or [64].

#### 3.2.7. Learning-Based Methods

The two most popular groups of anomaly detection methods that are based on widely understood learning, that exceed the above-mentioned groups, are active learning [65] and deep learning [66]. In the case of active learning, precisely selected unlabelled samples are taken into consideration to be manually labelled in the first order. Such an approach helps to improve the existing models with the update based on critical (boundary) samples [67]. Deep learning methods, on the other hand, try to build a model on unlabelled data on the basis of labelling provided by typical anomaly detection methods [68,69].

## 4. Anomaly Detection Module Environment

The anomaly detection module is the Python software was used to detect anomalies in network traffic in pseudo-real time. It runs in batch mode. The input data are taken directly from the network interface to which a copy of the traffic from the monitored (sub) network is redirected. The results of the analysis are saved as CSV files; it is also possible to send the detected anomalies to SIEM. The statistical anomalies in time and volume are assumed to be true outliers from dominant normal network traffic.

The primary element of the anomaly detection module is a Sniffer that collects and analyses packets in given time windows. It has two modes:

- online: based on the Scapy AsyncSniffer [70], collecting and processing packets from a network, used for pseudo-real-time monitoring;
- offline: reading CSV files, previously created by the online mode. Typically used for tuning the algorithms or reviewing historical traffic with other anomaly detection methods.

The first step is establishing a feature vector that is extracted from packets. This vector may contain any of the TCP/IP headers. Afterwards, aggregation is carried out in a given time window due to:

- source/destination IP address;
- pairs of IP addresses;
- IP address and port pairs.

One Sniffer instance can simultaneously run many feature vector/aggregation pairs and save results to a CSV file or send them directly to anomaly detection algorithms.

### 4.1. Implemented Anomaly Detection Methods

The anomaly detection module implements two well-known methods of anomaly detection: the first of them is the robust kernel-based local outlier detection method [15] and the second is the variational auto-encoding method [71]. As ADM is written in Python it can be extended by new anomaly detection methods at any time. The presented solution is also easy to configure even without programming knowledge; it is based on running a command-line program with input parameters defined in a text file.

#### 4.1.1. Robust Kernel-Based Local Outlier Detection (RKOF)

RKOF (robust kernel-based local outlier detection) is a factor that ranks objects according to their atypicality [15]. Instead of LOF [14] the method is based on the weighted neighbour density at the point rather than on the average neighbour density. As the authors claim, such a modification improves the possibility of outliers detection even if the number is comparable to the typical object number in some neighborhood.

The interpretation of obtained factors shows the following: observations with RKOF<1 should not be considered outliers, while the other (with RKOF>1) seem to not be typical observations.

#### 4.1.2. VAE

AE (autoencoder) and VAE (variational autoencoder) are anomaly detection methods based on neural networks. The network “learns” to reproduce the training data provided to it, assuming that they are typical values for the system, aiming to minimize the error in data reproduction. The reconstruction error is the arithmetically calculated difference between the output and input data of the model. In a running system, the trained model processes incoming data. In case the predefined reproduction error threshold is exceeded, an anomaly is detected.

### 4.2. Model Construction

The prediction model used the TensorFlow technology, which included the Keras API extension. The featured model contains multiple layers of neural networks.

The first layer is the normalization layer. To achieve a speed up of data processing (when compared to the sequential approach) normalization is performed on data integrated into tensors. All parameters in the input vector need to be individually normalized, so the mean, standard deviation, minimum, maximum, offset, and scale for each are stored in the tensors. With this solution and consideration of an additional parameter α indicating the speed of normalization adaptation to changing data, input tensors are normalized in parallel by the network.

The following layer is variation encoder. A particular encoder extract features from one-dimensional data. Each dense layer of neurons decreases its size in relation to the previous one, allowing for a reduction in the number of features. The obtained features are forwarded to distribution as a diagonal Gaussian, which delivers the results of encoding.

The task of the decoder as a final element of the network is to reconstruct the normalized input data. The results are acquired by moving the encoded data to the neural network, which most often is the mirror image of the encoder network, excluded the Gaussian sampling element.

To sum up, apart from the parameters denoted in the normalization layer, a number of neurons in each layer of the encoder, the activation function for each layer of the encoder, and a number of neurons in the layer between the encoder and decoder were used in the prediction model.

### 4.3. Training and Prediction

Considering that the featured network works not only as the variational autoencoder, but also includes a normalization layer, it returns not only the network reconstruction outcome but also the normalized input data. Therefore, it is possible to calculate the mean absolute error of the reconstruction, which is the anticipated result.

As a consequence of fitting the network with raw data, specified pipelines for training and testing the model were created. These functions take into consideration not only, as it often works, the input and output to compile the loss, but also both the previously mentioned model outputs.

Moreover, the sampling element used in the variation autoencoder remains the barrier that backpropagation cannot flow through; however, there exists a solution to this. Adding random noise to the sampling equation maintains the stochastic characteristic of the element and lets backpropagation through it.

The last, but not least, part of the training process is fitting the standardizer to decrease the unwanted differential in the returned information about the reconstruction error. This element will not only standardize the results in the prediction process but its will also slowly update the standardization parameters after each prediction time window.

### 4.4. Data Learning Selection

Due to the processing of large amounts of data, there is a concern about excessive memory or CPU use during training. Therefore, a selection system has been applied that limits the volume of data collected for training the model. The implementation is based on the publication “Catch It If You Can: Real-Time Network Anomaly Detection With Low False Alarm Rates” [72]. For the previously given time in which the collecting training data will occur, digitized time slices are determined to be gathered for training purposes. Time slice data are collected over the entire length of the predefined time window, with a greater density at the end of the window to emphasize the greater volume of more recent data.

### 4.5. Processing Time Window

Time window processing with the VAE model is not trivial, requiring the organization of new learning models and continuous prediction with the current model. Thus, the use of the autoencoder does not affect the performance of other system components, it is placed in a separate process. Thanks to this, the autoencoder process conducts training on itself and provides detection of the anomaly in incoming data. However, the superior class responsible for supervision over detection can collect training data and process new data by sending them to the autoencoder. The master class will create a new autoencoder process, training with collected data as fast as a new training dataset is gathered, while the training process will not end, anomaly detection will continue using the existing autoencoder. When the new model completes the learning stage, the master class will terminate the current autoencoder process and detect anomalies using the newly learned model.

## 5. Experiments

The experiments were planned to be carried out in an extended version of the virtual environment mentioned in [73]. However, the number of simulated workstations was increased to two attacking systems and four victims. The following subsections describe all experimental components in a more detailed way.

### 5.1. Hardware and Network Background

For the purpose of carrying out experiments on real network traffic, it became necessary to include artificial points (attackers and victims) in a physical network. As was already mentioned, these elements were virtual servers: two attackers and four victims. To assure as close as possible nature of attacks, the attackers and victims were ran on two different physical servers. Moreover, the ADM was ran on the third physical server.

**Figure 1:**
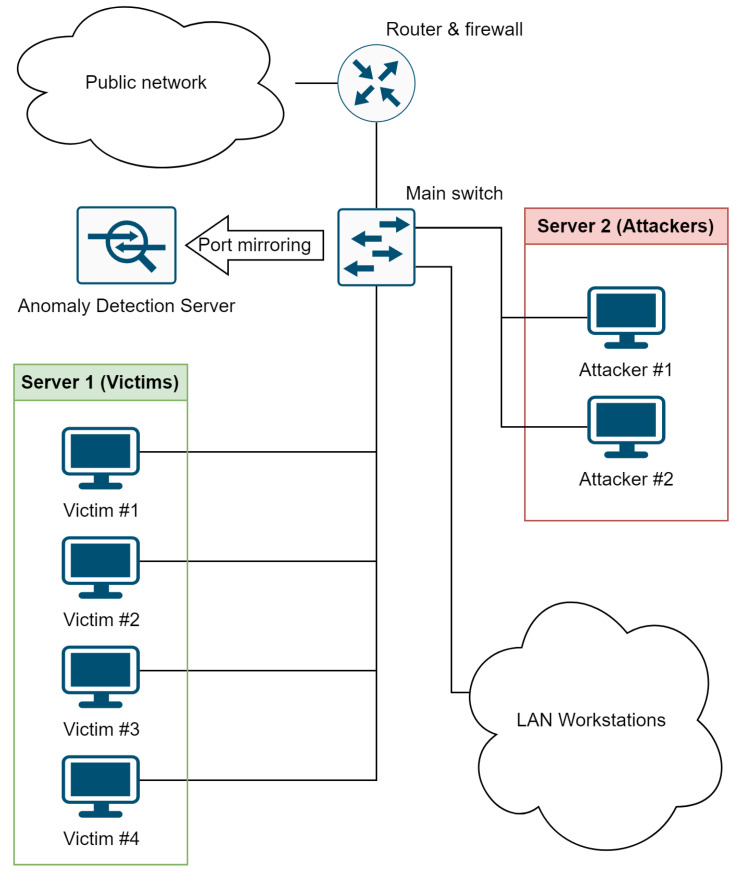
Network architecture scheme showing the placement of the anomaly detection server on the background of the inner infrastructure, including LAN workstations, servers with virtual attackers and victims.

The whole experiment was ran inside a Łukasiewicz EMAG network and all machines were connected together via a main LAN switch. This gave us the possibility to mix the typical network traffic with the forced attack-containing traffic. For the purpose of gathering all network traffic (ingoing and outgoing) a mirror port of a main switch was used. The network configuration and traffic flows are presented in Figure 1.

### 5.2. Data Preparation—Attack Script

As was already mentioned above, the experiment environment contained six virtual machines: two attackers and four victims. What is worth mentioning that the victim machines were ran using different operating systems: Ubuntu, Windows, and Kali Linux (both attackers were Kali Linux).

The goal of the experiment was to check the anomaly detection techniques to detect attacks performed within the network. It is much more reliable to evaluate anomaly detection techniques when the anomaly’s present location is known. In such a case the results may be evaluated as a binary classifier performance.

**Figure 2:**
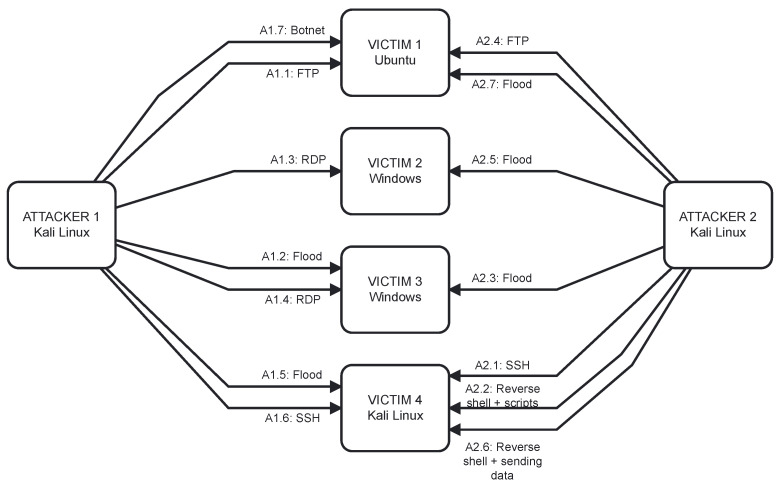
Diagram of the performed attacks presenting two attackers and four victims as well as planned attacks.

In our approach we decided to prepare an attack script that implies what kind of attacks will be run from which attacker on which victim. The schedule contained a variety of attacks and the scheme is presented in Figure 2.

The time aspect of each attack performance is described in the following subsection.

### 5.3. Collected Data Pre-Processing

**Figure 3:**
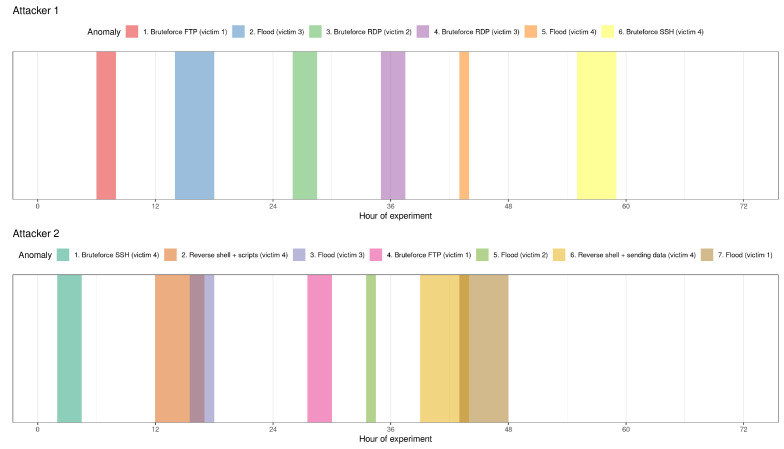
Graphic presentation of the moments of the attacks and their durations (for each attacker separately).

The experiment was conducted using the scenario described in Section 5. Due to technical reasons, the botnet attack on victim 1 did not take place. Additionally, we observed some unexpected traffic between attacker 2 and victim 4. For a better assessment of the ADM system, we decided to clean the analysed dataset and remove non-planned actions between the hosts. During further analysis, we identified a large number of flows directed towards the broadcast hosts. Thus, three broadcast hosts with the biggest number of flows were also removed from the analysed dataset. Finally, these two approaches to the data were examined, called, pre-processed and broadcast, respectively. The final attack timeline is presented in Figure 3.

The attacks were performed at different times of the day during the work week. The first attack took place in the afternoon. It can be noted that intrusions were executed in several combinations—only one attack in a given time and two or three overlapping attacks.

### 5.4. Results

Anomaly detection was performed with the ADM tool and two methods with different parameters. For the RKOF method, we set the number of neighbours to be equal to 100 and the window length equal to 10, 20, 30, and 60 min. For the VAE method, we considered three window lengths and connected them with the batch size and batch number. For a window length equal to 60 min, the batch size was 3 and the batch number was 20. In the case of 120 min they were 5 and 12, and for 180 min these parameters were set as 3 and 30, respectively. In all summaries presented in this paper, the window length is presented next to the name of the method, e.g., RKOF10.

Every row of collected data contains a single flow—aggregated for every minute of network traffic statistics between two hosts, such as the number of packets or port flags. Apart from the IP addresses and timestamps, the input dataset contains 121 attributes. The pre-processed dataset has 230,664 flows and the broadcast dataset has 81,027. The number of ground-truth anomalies (flows that occur at the time and between hosts specified in the scenario) in both cases was the same, 4371. Table 1 contains the number and percentage of detected anomalies.

The analysed methods usually report more anomalies than those arising from conducted attacks. It can be justified by the fact that apart from artificial intrusions there was normal network traffic during the whole experiment. For the pre-processed dataset, the lowest percentage of anomalies was observed for RKOF60 while the highest was for VAE120. In the case of the broadcast dataset, the RKOF60 also had the lowest percentage of anomalies but RKOF20 had the highest value. Based on the obtained results, we calculated classification quality measures (see Table 2).

The assessment of the results was based on three measures: balanced accuracy, sensitivity, and specificity. The best results measured by balanced accuracy were achieved with the RKOF30 method and the pre-processed dataset. In this case, the sensitivity was 66% which means that only that many anomalies were correctly identified. On the other hand, specificity in all the considered cases was very high. Based on the results in a Table 2, we could say that the ADM tool poorly detected true anomalies in the data. To verify the intrusion detection ability, we conducted additional calculations related to attacks only (Table 3).

The results in Table 3 show that the effectiveness of the method depends on its parameters and the type of attack. Most of the RKOF variants detected brute-force attacks with very high accuracy. In particular, brute-force SSH was only detected by RKOF methods and for broadcast data. In turn, VAE methods could better detect some flood intrusions. For the reverse shell, it is important to remove broadcast addresses, and then the RKOF methods can identify such a kind of attack. On average, the best results for the pre-processed data were obtained with the RKOF30 method. For broadcast data, RKOF30 performed the best based on the mean value and RKOF60 based on the median statistic. The conclusion from the conducted analysis is the link between the method and type of attack. Thus, in the next step, we proposed an ensemble approach that combined all the considered methods in anomaly detection.

**Figure 4:**
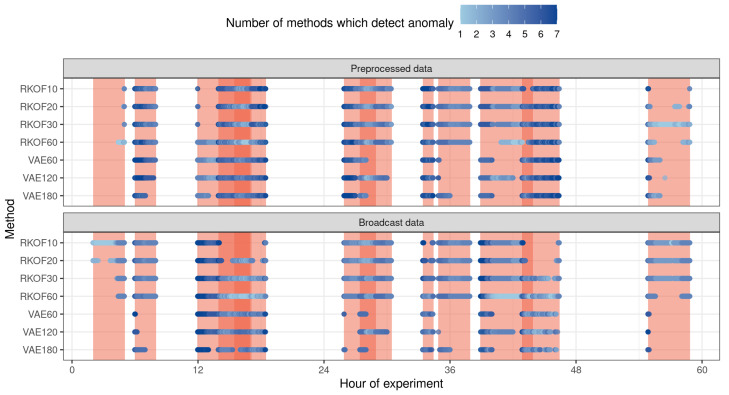
Anomalies in the traffic (vertical bars) and correctly recognized anomalies (points) for each model separately; the more intense the colour of the point, the more models recognized the anomaly.

Every point in Figure 4 presents an anomaly detected by any of the considered methods and the colour shows the number of methods that detect this intrusion. Red stripes in the background illustrate the time ranges of attacks and the intensiveness of colour shows the number of these attacks in a given time. It can be noted that these methods are complementary to each other and their combination is justified. We considered all possible combinations of these methods and calculated the quality measures. The ten best results according to the balanced accuracy measure are presented in Table 4.

It can be seen that the ensemble approach had a better ability for anomaly detection than the single-method approach. A combination of the different methods allows us to obtain balanced accuracy above 0.9 for pre-processed data and above 0.85 for broadcast data. Values of sensitivity and specificity are also higher.

Furthermore, the analysis with combined methods was conducted only within planned attacks. For each method combination, the percentage of correctly detected flows was calculated and the obtained results were averaged by method combination. If there were the same results for a few approaches, then the simplest—one with the lowest number of utilized methods—was chosen. The results for the five best ensembles for each dataset are presented in Table 5.

For selected intrusions, we could achieve 100% efficiency. In the case of pre-processed data, the best method combined with RKOF10, RKOF30, RKOF60, and VAE180 had 100% accuracy for 6 out of 13 intrusions. The most difficult attack to detect was the A2.1: Brute-force SSH, only possible to detect in only 19.61% of the flows labelled as anomalies. Another interesting case is the A1.2: Flood, where the percentage of detected anomalies was equal to 100% only if the RKOF10 was included.

Removing the broadcast addresses allowed us to obtain a higher overall accuracy. For the RKOF10, RKOF30, RKOF60, VAE60, and VAE180 ensemble obtained the lowest score for the A1.5, A2.2, and A2.7 attacks. Detection of the A2.1: Brute-force SSH intrusion was problematic for the pre-processed data as it was detected with 99.45% accuracy.

To sum up, we proposed an approach for anomaly detection based on two pillars, appropriate data preparation and the combination of different methods for anomaly detection. The analysis of two datasets showed that removing the broadcast addresses improved the recognition efficiency of various kinds of attacks. We observed that intrusion detection was strictly connected with the used method and separately these methods perform rather poorly. The combination of the RKOF method with various window lengths and variational autoencoder allowed anomalies detection with almost 97% accuracy measured as an average over the conducted attacks. We observed that in the final solution, only five out of seven investigated approaches were used which shows that greedy ensembling led to worse results and there is a need to look at optimal combinations.

## 6. Conclusions and Further Works

In this paper, a complete and configurable tool for the improvement of network security was presented. ADM was designed as an addition to existing cybersecurity solutions that runs in real time. The ADM tries to detect non-typical network traffic descriptions without trying to explain them. ADM was developed as a part of a wider project called RegSOC [6,7].

The presented ADM has the ability to work in two modes:

- online: such a mode is dedicated to the application phase when the anomaly detection models are trained sufficiently to detect anomalies in a network traffic description and it is possible to generate reports summing up the detected anomalies (to be further proceeded by a regional security operator);
- offline: a mode for tuning an anomaly detection method with data of known anomaly positions; such a mode helps to tune anomaly detection methods much faster than in the online (almost real-time) mode.

Apart from the above-mentioned two modes of operation, ADM provides two methods of anomaly detection—RKOF and VAE—and each of them may operate independently on separate network traffic descriptions. Moreover, it is possible to use several of them (e.g., several RKOF-based models with different parameters) at the same time. Thanks to this, it is possible to develop an ensemble-based voting model pointing to whether we there is an anomaly or not. The suggested exhaustive strategy of the best model of anomaly detection search should be easily extended to some heuristic search, such as climbing down.

The above-described features help to conclude the strongest points of the ADM:

- the ADM is an autonomic and complete solution, based on well-known methods of anomaly detection, also equipped with strategies to train and tune;
- the ADM was developed as an additional tool to complement other network monitoring systems, e.g., ElasticSearch [74];
- it is expected that the ADM may detect zero-day attacks whose signatures remain unknown for signature-based systems because the ADM is a stream-based system;
- thanks to this, it is possible for the ADM to detect occurrences that are not detected by other tools, i.e., signature-based;
- the methodology of the tuned model is also provided with the module.

In conclusion, the ADM may be an additional tool to improve network security. The gathered data are publicly available at http://ibemag.pl/pl/szczegoly-projektow#RegSOC (in the Available Datasets section). Moreover, the tool is still being developed to provide more anomaly detection method implementations, and it is still bing tested on new attack scenarios. It is expected that these data analysis results will be published soon.

## Figures and Tables

**Table 1:** A comparison of the number of samples recognized as anomalies and the percentage of actual anomalies.

Method	Number of Anomalies	Percentage of Anomalies
Pre-processed data		
RKOF10	8262	3.58
RKOF20	5840	2.53
RKOF30	6252	2.71
RKOF60	4113	1.78
VAE60	8060	3.49
VAE120	22,703	9.84
VAE180	20,156	8.74
Broadcast data		
RKOF10	7466	9.21
RKOF20	10,715	13.22
RKOF30	9208	11.36
RKOF60	4929	6.08
VAE60	6614	8.16
VAE120	10,629	13.12
VAE180	9514	11.74

**Table 2:** Classification performance measures for several models of anomaly detection.

Method	Balanced Accuracy	Sensitivity (TPR)	Specificity (TNR)
Pre-processed data			
RKOF10	0.7337	0.4944	0.9730
RKOF20	0.7598	0.5351	0.9845
RKOF30	0.8251	0.6651	0.9852
RKOF60	0.7438	0.4962	0.9914
VAE60	0.6781	0.3844	0.9718
VAE120	0.6700	0.4319	0.9080
VAE180	0.7049	0.4894	0.9204
Broadcast data			
RKOF10	0.7575	0.5793	0.9356
RKOF20	0.7390	0.5845	0.8936
RKOF30	0.7976	0.6767	0.9185
RKOF60	0.7777	0.5864	0.9691
VAE60	0.5751	0.2237	0.9265
VAE120	0.5441	0.2146	0.8736
VAE180	0.6244	0.3528	0.8960

**Table 3:** Percentage of flows within attacks labelled as an anomaly.

Attack	RKOF10	RKOF20	RKOF30	RKOF60	VAE60	VAE120	VAE180
Pre-processed data							
A1.1: Brute-force FTP	100.00	80.83	100.00	87.08	51.67	51.25	40.83
A1.2: Flood	53.72	58.78	65.96	37.77	60.64	63.83	56.12
A1.3: Brute-force RDP	99.45	99.72	80.66	34.81	20.44	70.17	22.93
A1.4: Brute-force RDP	100.00	100.00	100.00	72.65	33.43	1.66	0.55
A1.5: Flood	14.75	36.07	87.70	41.80	75.41	86.07	87.70
A1.6: Brute-force SSH	2.08	17.50	94.58	30.83	9.79	7.71	2.29
A2.1: Brute-force SSH	1.10	2.76	0.55	19.61	0.00	0.00	0.00
A2.2: Reverse shell + scripts	0.32	0.96	0.64	66.99	40.71	76.60	76.28
A2.3: Flood	47.24	50.28	68.23	50.83	63.26	65.19	60.77
A2.4: Brute-force FTP	50.55	49.45	66.57	82.87	19.89	19.89	86.19
A2.5: Flood	53.28	71.31	75.41	100.00	81.15	83.61	90.98
A2.6: Reverse shell + sending data	49.69	50.93	48.45	24.33	27.22	27.22	76.70
A2.7: Flood	71.70	86.08	90.80	53.30	79.01	80.66	88.21
Mean	49.53	54.21	67.66	54.07	43.28	48.76	53.04
Median	50.55	50.93	75.41	50.83	40.71	63.83	60.77
Broadcast data							
A1.1: Brute-force FTP	100.00	100.00	100.00	90.42	28.33	2.50	8.33
A1.2: Flood	2.13	23.67	50.53	58.78	30.59	39.63	40.43
A1.3: Brute-force RDP	100.00	100.00	78.45	32.60	3.87	0.55	0.00
A1.4: Brute-force RDP	100.00	100.00	100.00	71.27	33.15	0.00	0.28
A1.5: Flood	9.84	24.59	42.62	40.16	59.02	80.33	81.15
A1.6: Brute-force SSH	99.17	94.58	94.58	37.29	1.67	1.67	0.83
A2.1: Brute-force SSH	99.45	34.81	23.48	19.89	0.00	0.00	0.00
A2.2: Reverse shell + scripts	76.28	75.64	75.64	78.21	35.90	54.17	76.28
A2.3: Flood	2.21	35.08	52.49	75.69	20.99	20.44	25.97
A2.4: Brute-force FTP	51.38	51.38	96.13	85.08	19.89	19.89	85.36
A2.5: Flood	12.30	31.97	52.46	99.18	57.38	68.85	87.70
A2.6: Reverse shell + sending data	50.10	50.31	51.75	55.67	24.12	23.09	54.85
A2.7: Flood	5.19	14.15	47.64	54.72	31.60	38.68	59.43
Mean	54.46	56.63	66.60	61.46	26.65	26.91	40.05
Median	51.38	50.31	52.49	58.78	28.33	20.44	40.43

**Table 4:** Top 10 method combinations arranged by balanced accuracy.

Methods	BACC	Sens.	Spec.
Pre-processed			
RKOF20, RKOF30, RKOF60, VAE60	0.9162	0.8829	0.9495
RKOF10, RKOF30, RKOF60, VAE60	0.9152	0.8895	0.9409
RKOF10, RKOF20, RKOF30, RKOF60, VAE60	0.9140	0.8895	0.9384
RKOF10, RKOF30, RKOF60	0.9138	0.8650	0.9626
RKOF10, RKOF20, RKOF30, RKOF60	0.9124	0.8652	0.9595
RKOF30, RKOF60, VAE60	0.9075	0.8595	0.9554
RKOF20, RKOF30, RKOF60	0.9071	0.8403	0.9740
RKOF10, RKOF30, RKOF60, VAE180	0.9026	0.9165	0.8887
RKOF20, RKOF30, RKOF60, VAE180	0.9016	0.9053	0.8978
RKOF10, RKOF20, RKOF30, RKOF60, VAE180	0.9013	0.9165	0.8861
Broadcast			
RKOF10, RKOF30, RKOF60	0.8893	0.9410	0.8376
RKOF10, RKOF60	0.8876	0.8673	0.9078
RKOF10, RKOF60, VAE60	0.8792	0.9170	0.8414
RKOF10, RKOF60, VAE180	0.8787	0.9405	0.8168
RKOF30, RKOF60	0.8749	0.8545	0.8954
RKOF10, RKOF30, RKOF60, VAE60	0.8665	0.9570	0.7761
RKOF10, RKOF20, RKOF30, RKOF60	0.8655	0.9410	0.7901
RKOF10, RKOF30, RKOF60, VAE180	0.8646	0.9737	0.7555
RKOF10, RKOF60, VAE60, VAE180	0.8643	0.9430	0.7856
RKOF10, RKOF60, VAE120	0.8626	0.9286	0.7966

**Table 5:** Percentage of flows within attacks labelled as an anomaly for method ensembles.

Pre-Processed Data					
Attack\Methods	RKOF10, RKOF30, RKOF60, VAE180	RKOF20, RKOF30, RKOF60, VAE60, VAE120, VAE180	RKOF20, RKOF30, RKOF60, VAE120, VAE180	RKOF20, RKOF30, RKOF60, VAE60, VAE180	RKOF20, RKOF30, RKOF60, VAE180
A1.1: Brute-force FTP	100.00	100.00	100.00	100.00	100.00
A1.2: Flood	99.73	96.01	96.01	95.21	90.69
A1.3: Brute-force RDP	100.00	100.00	100.00	100.00	100.00
A1.4: Brute-force RDP	100.00	100.00	100.00	100.00	100.00
A1.5: Flood	98.36	98.36	98.36	98.36	98.36
A1.6: Brute-force SSH	94.58	94.58	94.58	94.58	94.58
A2.1: Brute-force SSH	19.61	19.61	19.61	19.61	19.61
A2.2: Reverse shell + scripts	87.50	87.50	87.50	87.50	87.50
A2.3: Flood	100.00	97.24	96.96	96.96	95.86
A2.4: Brute-force FTP	100.00	100.00	100.00	100.00	100.00
A2.5: Flood	100.00	100.00	100.00	100.00	100.00
A2.6: Reverse shell + sending data	98.97	98.97	98.97	98.97	98.97
A2.7: Flood	99.76	99.76	99.76	99.76	99.76
Mean	92.19	91.70	91.67	91.61	91.18
Median	99.76	98.97	98.97	98.97	98.97
Broadcast data					
Attack\Methods	RKOF10, RKOF30, RKOF60, VAE60, VAE180	RKOF10, RKOF30, RKOF60, VAE180	RKOF10, RKOF30, RKOF60, VAE60, VAE120	RKOF10, RKOF20, RKOF60, VAE60, VAE120, VAE180	RKOF10, RKOF20, RKOF60, VAE120, VAE180
A1.1: Brute-force FTP	100.00	100.00	100.00	100.00	100.00
A1.2: Flood	99.73	99.73	99.73	91.22	91.22
A1.3: Brute-force RDP	100.00	100.00	100.00	100.00	100.00
A1.4: Brute-force RDP	100.00	100.00	100.00	100.00	100.00
A1.5: Flood	86.89	86.89	86.89	86.07	86.07
A1.6: Brute-force SSH	99.17	99.17	99.17	99.17	99.17
A2.1: Brute-force SSH	99.45	99.45	99.45	99.45	99.45
A2.2: Reverse shell + scripts	87.18	87.18	87.18	87.18	87.18
A2.3: Flood	100.00	100.00	100.00	98.62	98.62
A2.4: Brute-force FTP	100.00	100.00	98.62	100.00	100.00
A2.5: Flood	100.00	100.00	100.00	100.00	100.00
A2.6: Reverse shell + sending data	100.00	100.00	100.00	100.00	100.00
A2.7: Flood	87.97	87.74	77.83	87.03	86.79
Mean	96.95	96.93	96.07	96.06	96.04
Median	100.00	100.00	99.73	99.45	99.45

## Data Availability

The gathered data are available through http://ibemag.pl/pl/szczegoly-projektow#RegSOC (in the Available Datasets section).
